# Polymorphic Ventricular Tachycardia Detected by a Smartwatch in a Patient With Recurrent Syncope

**DOI:** 10.1016/j.jaccas.2024.102606

**Published:** 2024-10-02

**Authors:** Yuval Avidan, Vsevolod Tabachnikov, Asaf Danon, Jorge E. Schliamser

**Affiliations:** a Department of Cardiology, Lady Davis Carmel Medical Center, Haifa, Israel; b Ruth and Bruce Rappaport Faculty of Medicine, Technion- Israel Institute of Technology, Haifa, Israel

**Keywords:** polymorphic ventricular tachycardia, smartwatch, vasospastic angina

## Abstract

The diagnosis of vasospastic angina remains challenging. It may present with syncope and life-threatening arrhythmia. Patients with syncope usually require automatic modes of recording. We describe a patient with recurrent syncope and polymorphic ventricular tachycardia documented by a smartwatch. Eventually, the arrhythmia was attributed to vasospasm and effectively managed with pharmacotherapy.

## Graphical Abstract

**Figure:**
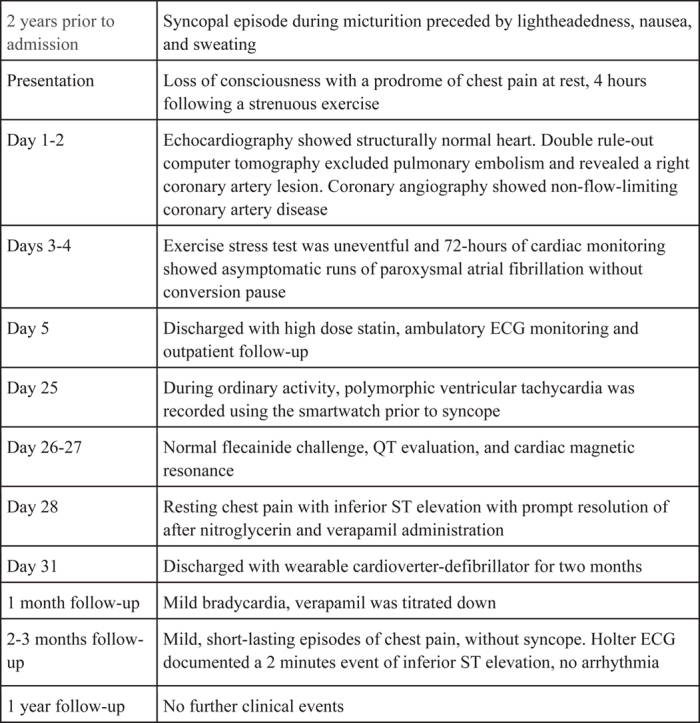

A 54-year-old man, a recreational athlete, presented following an unwitnessed syncope. The syncopal episode occurred 4 hours after biking and was preceded by chest pain. The results of physical examination were normal except for irregular heart sounds. An electrocardiogram (ECG) while he was asymptomatic showed atrial fibrillation, which spontaneously converted to sinus rhythm after several hours. ECG during sinus rhythm was unremarkable. Echocardiography demonstrated normal left ventricular function. The cardiac enzyme levels were minimally elevated (troponin 18 ng/dL; upper normal value 13 ng/dL), D-dimer was within normal limits. Lipid profile showed low-density lipoprotein of 131 mg/dL. Cardiac computed tomography angiography showed coronary atherosclerosis with a moderate right coronary artery (RCA) lesion. Next, coronary angiography verified a nonobstructive RCA lesion and normal left system. He was discharged on atorvastatin 80 mg, and a recommendation to perform ambulatory ECG monitoring; he also chose to use a smartwatch. Two weeks later he experienced another episode of syncope preceded by chest pain. He was able to record the beginning of the episode before loss of consciousness occurred and his finger was no longer pressed against the watch. The recording showed polymorphic ventricular tachycardia (PMVT) (Figure 1).

**Learning Objectives**

• To identify the potential utility of smartwatches in the diagnostic work-up of syncope with prodrome
• To recognize the role of smartwatches as patient-initiated monitoring tools for potential ventricular arrhythmias, which in turn could be of value in patients with suspected VSA

**Figure 1:**
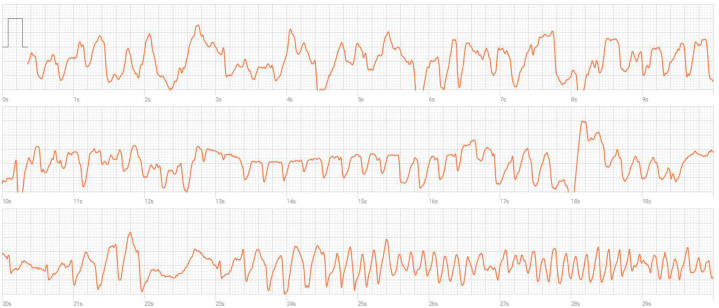
Smartwatch ECG Prior to Syncope Lead I electrocardiogram recording from a Samsung Galaxy Watch series 4, during a prodrome that included chest pain and dizziness. Immediately thereafter the patient lost consciousness for several seconds. The recording is notable for a run of polymorphic ventricular tachycardia.

## Past Medical History

The patient was a nonsmoker, took no medications, and did not describe substance abuse. There was no family history suggestive of sudden cardiac death or coronary artery disease. Notably, 2 years earlier he had experienced a syncopal episode with anamnesis suggestive of vasovagal mechanism.

## Differential Diagnosis

Possible differential diagnoses considered were causes of PMVT. The first step was to determine whether the arrhythmia was related to a long QT syndrome, either congenital, drug induced, bradycardia induced, or resulting from electrolyte imbalance. PMVT in the setting of those pathologic conditions is referred to as “torsade de pointes.” After the exclusion of long QT syndrome, other entities in the differential diagnosis include myocardial ischemia, Brugada syndrome, short QT syndrome, malignant early repolarization, and catecholaminergic polymorphic VT.^1^ Various forms of cardiomyopathies and cardiac sarcoidosis may also result in PMVT.^2^

## Investigations

**Figure 2:**
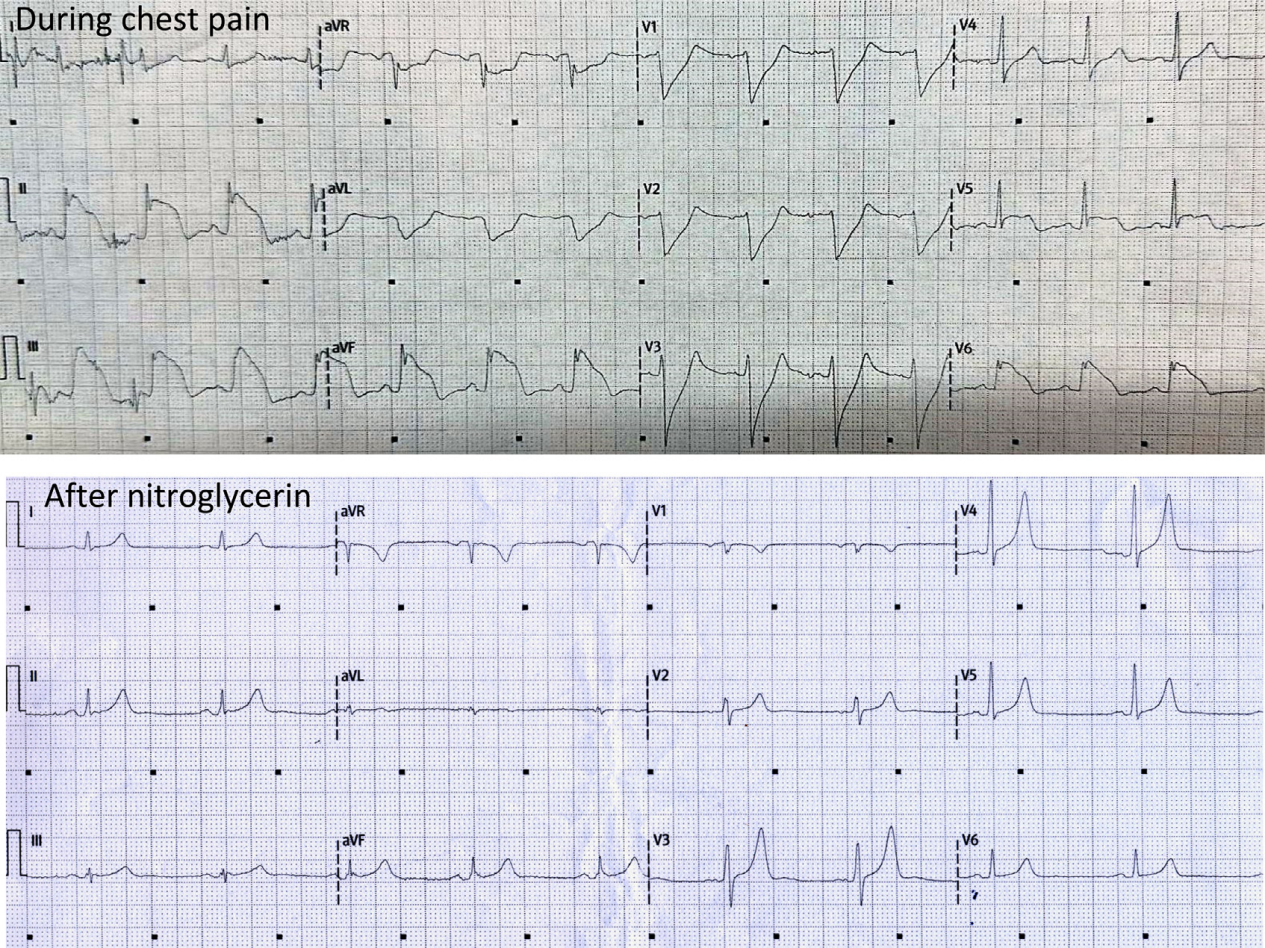
Inpatient ECG Recording (Top) Electrocardiogram (ECG) during admission while the patient was experiencing chest pain. (Below) ECG after treatment with nitroglycerin, showing resolution of the ST-segment elevation.

The results of 12-lead ECG and laboratory metabolic panel were unremarkable. The results of challenge with sodium channel blockers for Brugada syndrome and evaluation of the QT in supine and standing positions and after exercise testing were normal. Chest computed tomography results and level of serum angiotensin-converting enzyme were normal. CMR showed normal heart anatomy without signs of inflammation, ischemia, or fibrosis. Subsequently, resting chest pain recurred, accompanied by ST-segment elevation in inferior wall leads and reciprocal ST-segment depression (Figure 2). The symptoms and ECG changes promptly resolved after the administration of oral verapamil and sublingual nitroglycerin.

## Management

**Figure 3:**
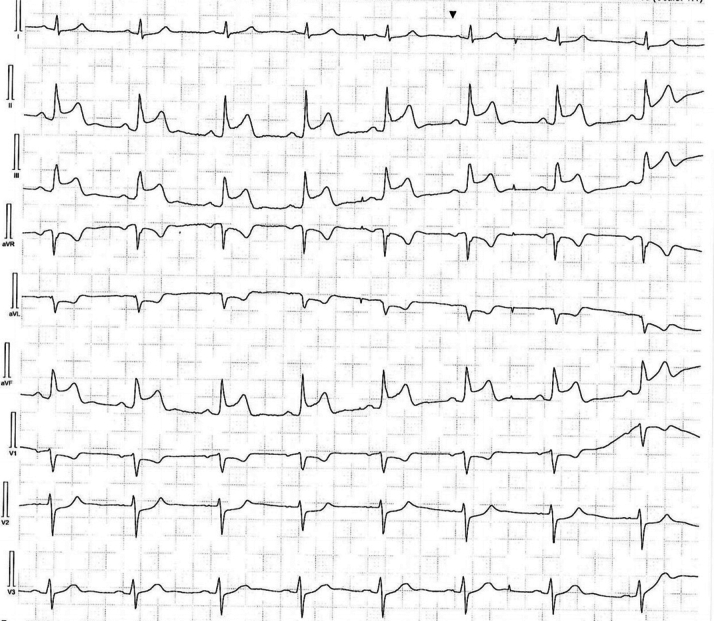
Outpatient Holter ECG Monitoring 12-lead Holter I electrocardiogram recording from a Samsung Galaxy Watch series 4, during an episode of mild chest pain without syncope or dizziness. The recording is notable for ST-segment elevation without arrhythmia.

High-dose verapamil (320 mg/day) was administered, and symptoms did not recur. Following a comprehensive discussion that emphasized the pros and cons of an implantable cardioverter-defibrillator (ICD), the patient opted for continuing medical treatment only. He was discharged with a wearable cardioverter defibrillator for 2 months. After 1 month, his medications were titrated down because of bradycardia (verapamil 240 mg/day). Two months after his last admission, the patient was mildly symptomatic, still having short infrequent episodes of mild chest pain. One of the episodes occurred during the use of 12-lead Holter ECG, lasted 2 minutes, and did show ST-segment elevation without arrhythmia, dizziness, or syncope (Figure 3). We advised him to use nitroglycerin spray immediately in case of such an event.

## Discussion

Vasospastic angina (VSA) is the consequence of myocardial ischemia caused by transient epicardial coronary spasm. Although VSA is a well-documented entity, it is perceived as an under-recognized cause of syncope.^3^ The most prominent features of this condition include chest pain during rest, ST-segment deviation, and prompt resolution with nitrates.^3^ However, one of the main diagnostic challenges is the need to link these ECG changes with the brief duration of symptoms.^4^

In 2015, a standardization of diagnostic criteria for VSA was proposed^5^ and has been adopted worldwide by various societies. In short, a “definitive VSA” could be established in the presence of nitrate-responsive angina either by coronary angiography with pharmacologic provocation and/or by ambulatory ECG. In clinical practice, intracoronary provocation testing has been restricted to specialized centers, and its unavailability poses a major pitfall.

Ambulatory ECG monitoring comes in different shapes and forms. Patients with syncope usually require automatic modes of recording such as external loop recorders or implantable cardiac monitors.^6^ External loop recorders are inconvenient and usually cannot be used for more than a few weeks. Implantable cardiac monitors are costly and require an implanting procedure. Smartwatches are becoming very popular, and the cost is decreasing.^7^ Their main advantage is their convenience. Some of the new devices combine photoplethysmogram sensors with the ability to record a single-lead ECG. This is done using 2 “leads,” the watch on 1 hand and the finger of the second hand pressed against the watch. The result is a signal resembling lead I of the ECG. These devices may be of value in diagnosing hemodynamically stable arrhythmias.^8^ However, smartwatch-based detection of ventricular arrhythmias such as PMVT is rare and has not been previously described in a patient with VSA. As our case demonstrates, this approach may play a valuable role in timely diagnosis and institution of adequate therapy in patients with syncope accompanied by a prodrome, including that caused by VSA.

Considering the management of VSA, smoking cessation and the use of calcium channel blockers with or without nitrates often ameliorate the symptoms and reduce recurrence.^3^ Whether ICD implantation is appropriate remains debatable. No randomized controlled trials have compared ICD implantation with medical therapy alone. The American College of Cardiology/American Heart Association guidelines divide their recommendations regarding ICD implantation in VSA patients who have survived sudden cardiac arrest. A recommendation at the level of Class IIa/Level of Evidence: B if sudden cardiac arrest recurs despite medical therapy, whereas a Class IIb/Level of Evidence: B recommendation for ICD implantation even before determining the effectiveness of medical therapy.^9^ In our case, given that as the vasospasm led to PMVT and syncope, one would argue that there is a justification for ICD. Nonetheless, no arrhythmia episodes were recorded after the initiation of pharmacotherapy.

Finally, although pharmacologic intervention did suppress the occurrence of arrhythmia and episodes of syncope, the patient experienced intermittent chest pain accompanied by ST-segment elevation on Holter ECG during the initial follow-up. A possible explanation is that individuals with coronary atherosclerosis exhibit heightened sensitivity to vasospasm. As evident in our case, the atherosclerotic lesion in the RCA, which was confirmed through angiography, aligns with the sites of ST-segment elevation observed on both the 12-lead ECG and the Holter ECG (Figures 2 and 3). It is plausible that the high-dose statin treatment resulted in stabilization of the atherosclerotic plaque, thereby diminishing the frequency of clinical events.

## Follow-Up

After 1 year of outpatient follow-up, there were no further clinical events.

## Conclusions

Inasmuch as the diagnosis of VSA remains challenging, physicians should increase their awareness of the existence of VSA and its elusive nature. This case displays the novel utility of A smartwatch-based diagnosis of hemodynamically unstable arrhythmia, eventually resulting in A time-sensitive diagnosis and the initiation of targeted therapy. It is plausible to expect this user-friendly technology to become an integrated part of the diagnostic work-up in the future.

## Funding Support and Author Disclosures

The authors have reported that they have no relationships relevant to the contents of this paper to disclose.
